# Evaluation of the contribution of fine-needle non-aspiration cytology to diagnosis in cases with pulmonary malignant lesions

**DOI:** 10.3906/sag-2104-363

**Published:** 2021-09-30

**Authors:** Şenay YILMAZ, AK Güntülü, Selma METİNTAŞ, Emine DÜNDAR, Muzaffer METİNTAŞ

**Affiliations:** 1 Department of Pulmonary Diseases, Faculty of Medicine, Eskişehir Osmangazi University, Eskişehir, Turkey; 2 Department of Pulmonary Diseases, Lung and Pleural Cancers Research and Clinical Center, Faculty of Medicine, Eskişehir Osmangazi University, Eskişehir, Turkey; 3 Department of Public Health, Lung and Pleural Cancers Research and Clinical Center, Faculty of Medicine, Eskişehir Osmangazi University, Eskişehir, Turkey; 4 Department of Pathology, Lung and Pleural Cancers Research and Clinical Center, Faculty of Medicine, Eskişehir Osmangazi University, Eskişehir, Turkey

**Keywords:** Fine-needle non-aspiration cytology, peripheral pulmonary lesion, lymph node cytology

## Abstract

**Background/aim:**

Fine-needle non-aspiration cytology (FNNAC) is an easy-to-apply, minimally invasive diagnostic method that contributes to the diagnosis and staging of lung cancer. FNNAC can be performed from peripheral lymph nodes as well as in peripheral lung lesions. This study aimed to evaluate the contribution of FNNAC performed from peripheral lesions or lymph nodes to diagnosis in patients with pulmonary malignant lesions.

**Materials and methods:**

FNNAC was applied from a peripherally located mass in the lung, chest wall lesion, or peripheral lymph node using a needle without an injector or active suction. The collected material was evaluated using the cytoblock method. The FNNAC accuracy was obtained by dividing the true positivity value by a number of needle biopsies performed. The 95% confidence interval of the obtained rate was also calculated.

**Results:**

The mean age of 56 patients, two female (3.6%) and 54 male (96.4%), was 63.9 ± 9.1 (38–80) years. FNNAC was performed from the peripheral lymph node in 48 patients, the peripheral pulmonary lesion in four, and the accompanying chest wall lesion in four. While true positivity was present in 42 patients, two patients had true negativity, and 12 had false negativity. In five of the 12 cases reported as false negative, the collected material was evaluated as insufficient, while the malignant diagnoses of the remaining seven cases were confirmed by other diagnostic methods. The diagnostic success of FNNAC was determined as 78.57% (95% CI: 65.56–88.41). FNNAC was more successful in diagnosis when performed from the peripheral lymph node compared to the peripheral pulmonary lesion (p=0.033).

**Conclusion:**

In cases with pulmonary lesions, FNNAC performed from the peripheral lymph node is an easy-to-apply, cost-effective, minimally invasive method with a high diagnostic success and low complication rate. It can be preferred for diagnosis, staging, and recurrence in malignant cases not suitable for advanced invasive procedures.

## 1. Introduction

Fine-needle non-aspiration cytology (FNNAC) is an easy-to-apply, minimally invasive method that contributes to the diagnosis and staging of cancer. This technique was first applied to thyroid and breast lesions in France in 1982 [1,2]. In the literature, this technique has also been described using other terms, such as ‘non-aspirated cytology’, ‘fine-needle sampling without aspiration’, ‘cytopuncture’, and ‘fine-needle capillary sampling’. The non-aspiration technique is successfully used in breast, thyroid, and other superficial lesions because it has several advantages, including ease of application, adequacy of cells due to less blood content, and easier interpretation of results [3,4]. There are many studies comparing FNNAC with the classical aspiration technique, and the former has gained popularity in the field due to better results [2,3].

FNNAC is a procedure based on the mechanism of spontaneous pulling of the sample into the needle hub without active suction through capillary pressure created by the direct insertion of the needle without an injector into the lesion [2,3,5]. In addition to being more safely applied, especially in thyroid, liver, and orbital lesions with bleeding tendency, it is also employed easily in lymph node metastases [5–9]. Using FNNAC, a diagnosis can be made not only based on the examination of peripheral pulmonary lesions but also superficial organs and lymph nodes [10]. In the current study, we aimed to evaluate the diagnostic success of FNNAC performed from the peripheral lesion or peripheral lymph node in cases with pulmonary lesions.

## 2. Material and method

### 2.1. Study group

Fifty-six patients, who had a peripheral pulmonary lesion, chest wall lesion, or peripheral lymph node underwent FNNAC between 2016 and 2019 were included in the study. The study was performed by retrospectively analyzing the prospectively recorded data. Before the procedure, the patients were informed, and their consent was taken. Ethical approval was obtained from the Ethics Committee of Eskisehir Osmangazi University (Approval number: 02.02.2021/10).

After clinical and radiological evaluations, the following diagnostic procedures were also undertaken as necessary according to the locations of the lesion: fiberoptic bronchoscopy (FOB), transthoracic fine-needle aspiration biopsy (TTFNA), endobronchial ultrasound-guided lymph node biopsy (EBUS), pleural fluid cytology (PFC), pleural biopsy, and excisional lymph node biopsy.

In addition to the pathology results, the accuracy of the FNNAC results was evaluated based on the clinical and radiological findings, as well as the reports of other invasive procedures in some patients. According to the FNNAC results, the patients were divided into two groups: accurately diagnosed group consisting of patients with true positivity and negativity of malignancies diagnosed by FNNAC and inaccurately diagnosed group consisting of cases with false negativity of malignancies, i.e. those in which FNNAC was not able to diagnose malignancies that were confirmed to be present by other methods.

### 2.2. FNNAC procedure

**Figure 1:**
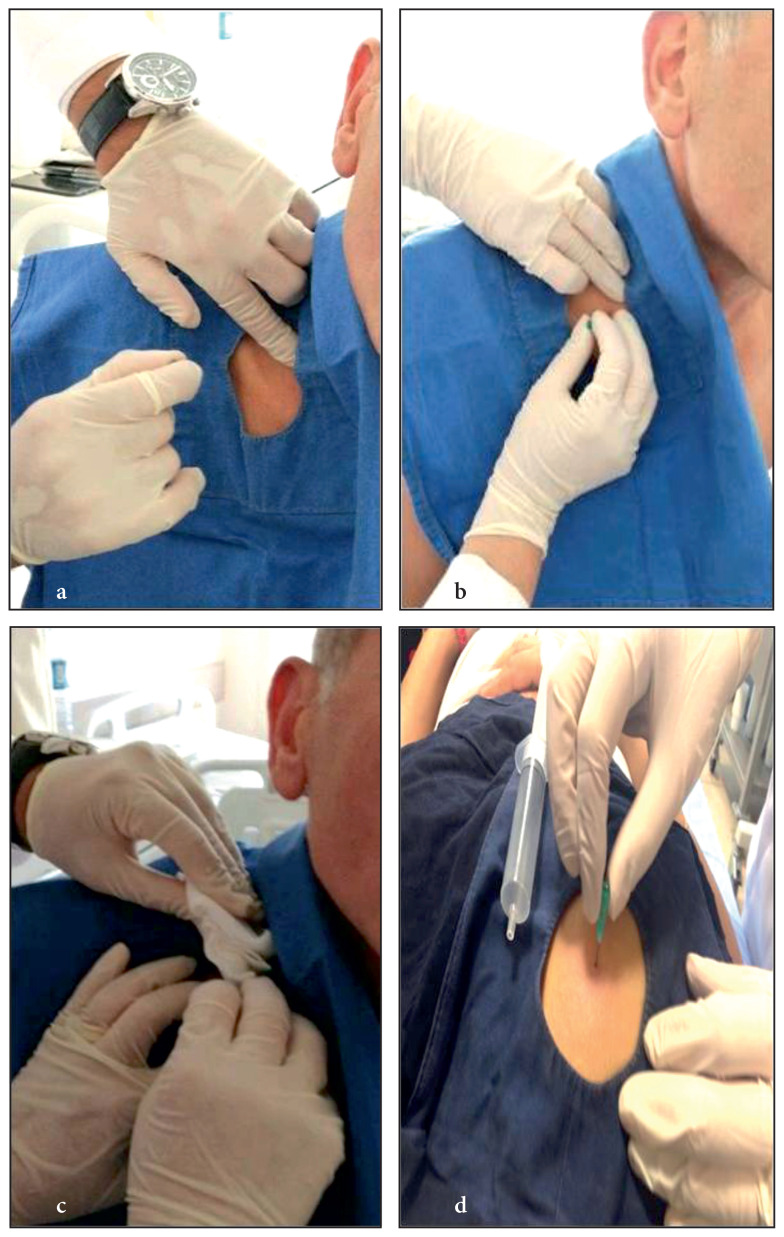
FNNAC performed from the supraclavicular lymph and peripheral pulmonary lesion. FNNAC performed from the supraclavicular lymph node (**1a-1c**). FNNAC performed from the peripheral pulmonary lesion (**1d**).

FNNAC was performed by inserting a needle without an injector into the lesion and quickly removing it and repeating this process several times, i.e. prompting the spontaneous pulling of the sample into the needle hub without active suction as shown in Figure 1a–1c and 1d. The patient was placed in a lying or sitting position according to the location of the lesion. The skin was disinfected with 10% polyvidone iodine, and local anesthesia was applied. After the lesion was detected between the thumb and index fingers of a hand, a 21-gauge needle without an injector was inserted into the lesion. In peripheral and chest wall lesions, the lesion site was checked, especially with thoracic ultrasonography, and FNNAC was applied from the marked area before the procedure. The needle was moved back and forth in different directions and at different angles within the lesion. Since there was no injector, no suction force was applied. After the needle was withdrawn, it was connected to a syringe filled with air. The syringe was then pushed to fill the tissue into the needle, and the content was placed in 10% buffered formol solution and transferred to the pathology department for analysis. If the sample taken is thought to be sufficient, one or two attempts may be sufficient. In our study, at least two and at most four attempts were made.

### 2.3. Cytopathological evaluation

**Figure 2:**
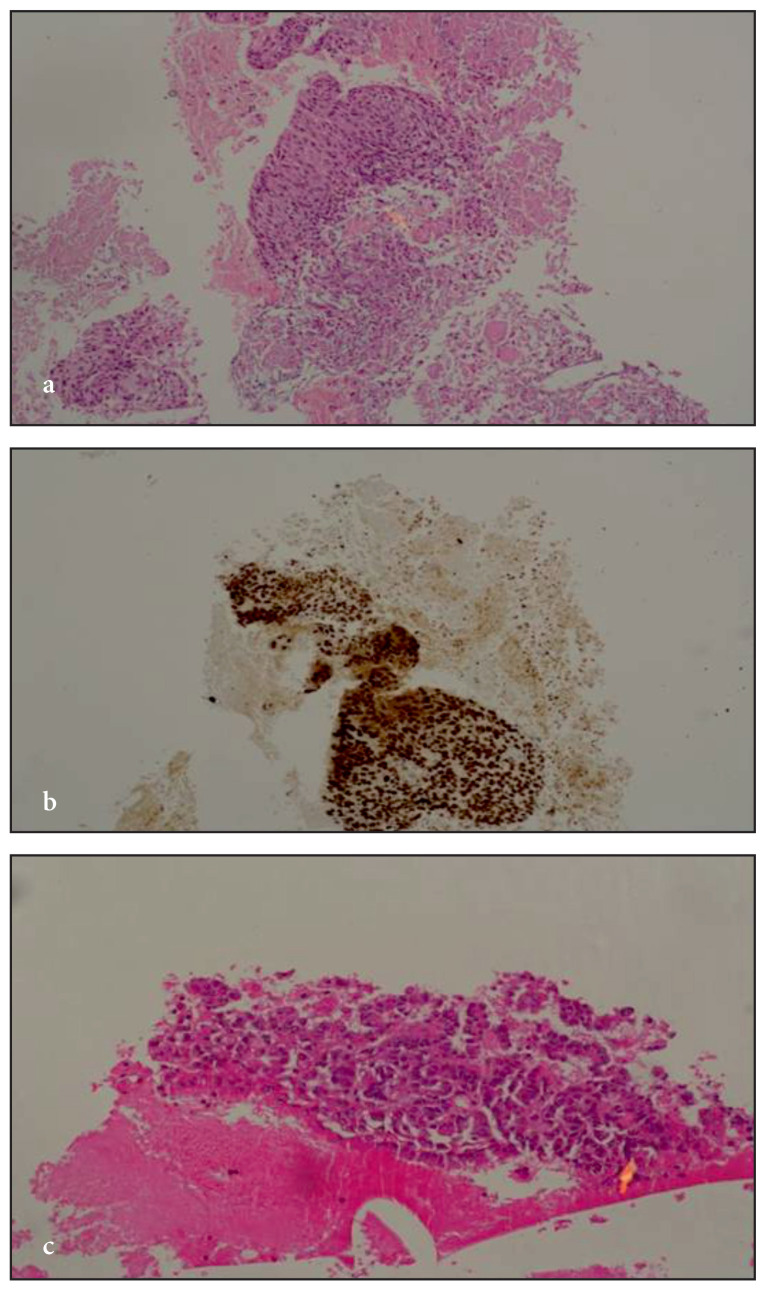

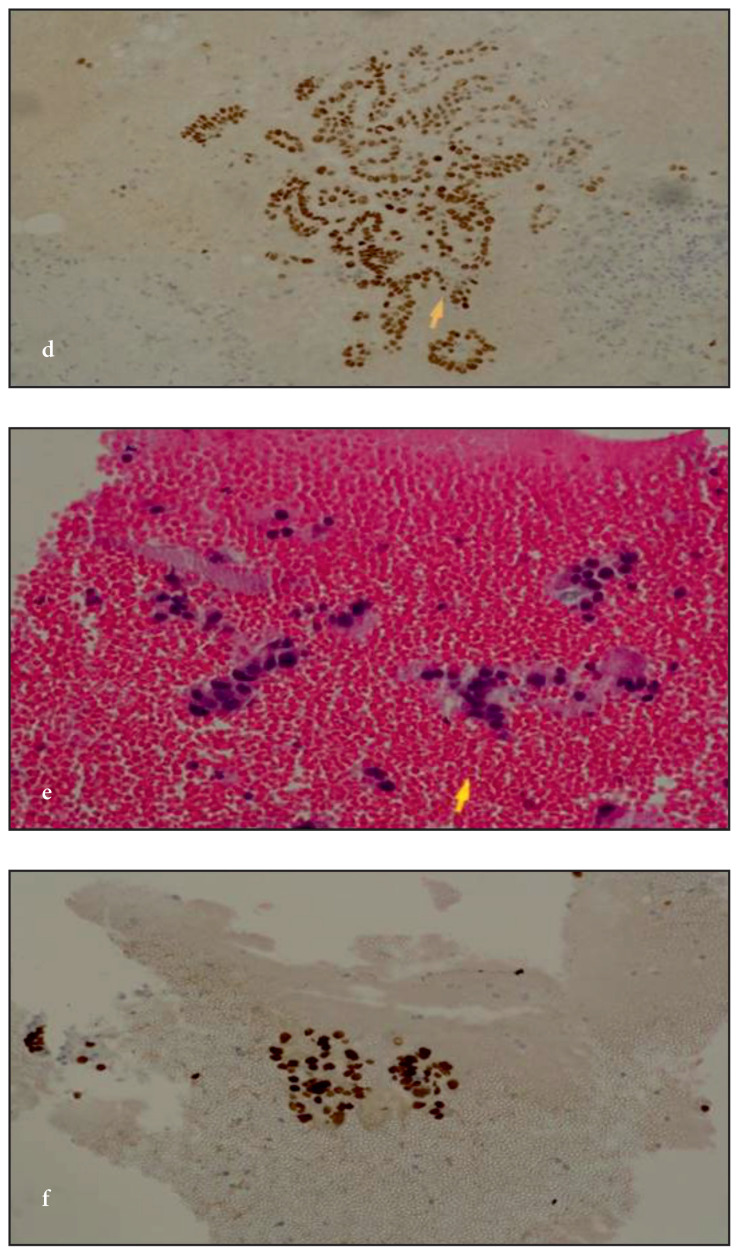
Histopathological and immunohistochemical findings of patients who underwent FNNAC. FNNAC performed from an axillary lymph node: squamous cell carcinoma. Tumoral tissue fragment consisting of atypical epithelial cells with squamous appearance in necrotic debris (H&Ex100) (**2a**), p63 immunohistochemical marker positivity in tumoral tissue fragment (p63x100) (**2b**). FNNAC performed from a supraclavicular lymph node: epithelioid malignant mesothelioma. Tumoral tissue fragment (H&Ex100) composed of atypical mesothelial cells with a trabecular pattern in blood mass (**2c**). WT-1 immunohistochemical marker positivity in atypical mesothelial cells (WT-1x100) (**2d**). FNNAC performed from a chest wall lesion: Pulmonary adenocarcinoma. Atypical epithelium arranged in a gland-like pattern within blood mass (**2e**). TTF-1 immunohistochemical marker positivity in atypical epithelial cells (TTF-1x200) (**2f**).

The cytoblock method was used for the pathological evaluation. The samples were evaluated histopathologically and immunohistochemically. The histopathological and immunohistochemical findings of three FNNAC samples taken from the axillary lymph node, supraclavicular lymph node, and chest wall lesion are shown in Figure 2a–2f.

### 2.4. Statistical analysis

Statistical analysis was performed using SPSS v. 15 (IBM Company, Armonk, NewYork) software package. The accuracy rate of the applied technique was obtained by dividing the true positivity value by the number of needle biopsies performed. Sensitivity was calculated from the ability of the method to distinguish patients from real patients, and the specificity was calculated from the ability of the method to distinguish healthy from real patients. The total correct diagnosis rate of the test gave the accuracy value of the test. The positive predictive value was calculated as the percentage of patients with a positive test who had the disease, and the negative predictive value was calculated as the percentage of patients with a negative test who did not have the disease. The number of false positive results for each true positive result of the diagnostic test was calculated as the positive likelihood ratio, and the number of false negative results for each true negative result of the diagnostic test was calculated as the negative likelihood ratio. The 95% confidence interval for each ratio was also calculated. Qualitative data obtained from the study were expressed as frequency and percentages, and quantitative data as mean, standard deviation, and extreme values (min, max). The X^2^ test was conducted to compare qualitative data. A p value of < 0.05 was considered statistically significant.

## 3. Results

Fifty-six patients with a peripheral pulmonary mass, a chest wall lesion, or a mass accompanied by a peripheral lymph node were included in the study. The characteristics of the patients regarding age, sex, diagnosis, and stage are shown in Table 1. While three (5.4%) patients had no smoking history, 41 (73.2%) patients were active smokers and 12 (21.4%) patients had quit smoking. The most common presentation symptom of the patients was cough (61.1%).

Using FNNAC, an accurate diagnosis was made in 44 of the 56 cases that underwent this procedure. Forty-two of these cases received a malignant diagnosis while the remaining two patients were detected to have benign diagnosis with FNNAC performed from the supraclavicular lymph node. Among the two benign cases, the benign diagnosis was confirmed as tuberculosis by an excisional biopsy of the supraclavicular lymph node in one and fibrinous pleuritis by a pleural biopsy in the other. FNNAC cytology of these two benign cases was reported as negative for malignancy. The distribution of lesions from which FNNAC was applied and the number of patients who were accurately diagnosed with FNNAC are given in Table 2.

While 34 of the 47 cases that underwent diagnostic FNNAC had true positivity, 11 had false negativity, and two had true negativity. Of the nine patients who were diagnosed with lung cancer and underwent FNNAC with the suspicion of progressive disease in the follow-up after treatment, eight were detected to have true positivity while one had false negativity. In total, 42 patients had true positivity, two had true negativity, and 12 had false negativity. Among the 12 patients, who were reported as false negative, the cell was evaluated as insufficient in five, of whom three were diagnosed with squamous cell carcinoma with FOB, one was diagnosed with squamous cell carcinoma using TTFNA, and one was diagnosed with mesothelioma using TTFNA. Table 3 shows the clinicopathological characteristics of the remaining seven patients who were reported to be negative for malignancy. No significant complications developed in the patients after the procedure. A mild subcutaneous hematoma developed in one case that underwent FNNAC, but no additional intervention was required.

**Figure 3:**
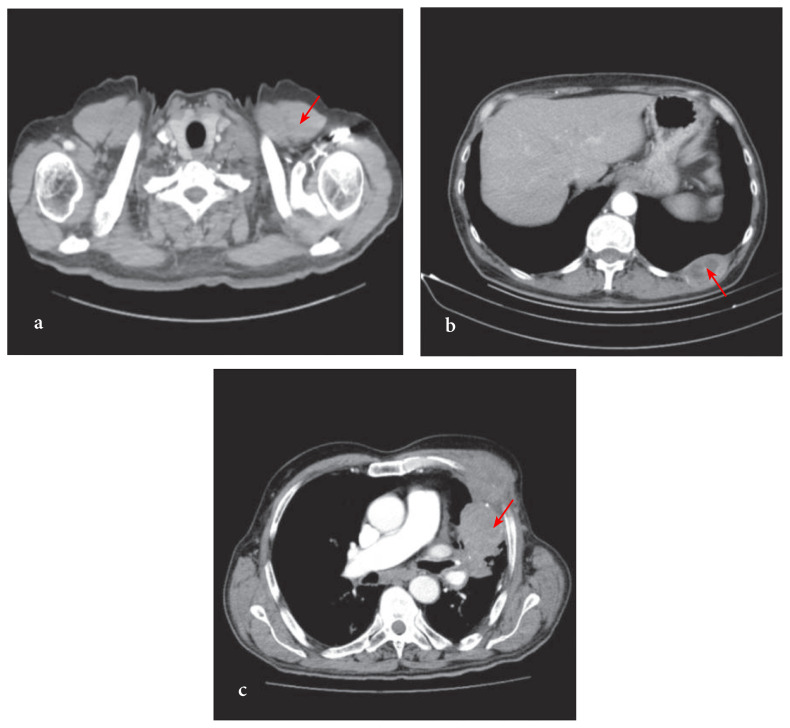
Radiological images of different lesions from which FNNAC was applied and diagnosis was made. FNNAC performed from a peripheral lymph node: squamous cell carcinoma (**3a**). FNNAC performed from a chest wall lesion: pulmonary origin adenocarcinoma (**3b**). FNNAC performed from a peripheral lesion invading the chest wall: epithelioid malignant mesothelioma (**3c**).

The comparative accurate diagnosis rates of FNNAC performed from the peripheral lymph node and peripheral pulmonary lesion are given in Table 4. It was observed that FNNAC was more successful in diagnosis when performed from the peripheral lymph node compared to when it was performed from the peripheral pulmonary lesion (X^2^= 4.52, p = 0.033). The radiological images of the different lesions from which FNNAC was applied are shown in Figures 3a–3c.

Forty-eight of the 54 patients with a malignant final diagnosis were diagnosed with lung cancer. Histopathologically, the number of true positive and negative diagnoses of SCLC and neuroendocrine carcinoma was 17 (100%), while there were no false-negative diagnoses. According to the same analysis, the group consisting of adenocarcinoma, squamous cell carcinoma, NSCLC, and non-neuroendocrine poorly differentiated carcinoma cases were detected to have 21 (72.4%) true positive and negative diagnoses, while the remaining eight (27.6%) had false-negative diagnoses. One case was reported as mixed-type lung cancer (adenocarcinoma + SCLC), and one was evaluated as clinical-radiological lung cancer not included in the group. The rate of FNNAC obtaining an accurate diagnosis was found to be statistically significantly higher in patients diagnosed with SCLC than those diagnosed with NSCLC (X^2^= 6.497, p = 0.019). Thus, for FNNAC, the sensitivity was determined as 77.78% (95% CI: 64.40–87.96), specificity as 100% (95% CI: 15.81–100.00), negative likelihood ratio (LR-) as 0.22, positive predictive value as 100%, negative predictive value as 14.2% 95% CI: 9.19–21.54), and diagnostic accuracy rate as 78.57% (95% CI: 65.56–88.41).

## 4. Discussion

FNNAC allows for a diagnosis in peripheral pulmonary lesions, as well as superficial organs and lymph nodes. In our study, the diagnostic success of FNNAC was found to be 78.57%, and it was observed that FNNAC performed from the peripheral lymph node was more successful in diagnosis than the procedure performed from the peripheral lung lesion. Our results support the idea that this method with a very low complication rate and cost effective can be preferred in diagnosis, staging and recurrence determination in malignant cases that are not suitable for more invasive procedures.

In the literature, while diagnostic aspiration applications have been described in many organs and peripheral lymph nodes, there are only limited studies concerning the use of FNNAC in patients with pulmonary lesions, peripherally localized lesions, chest wall invasion, or peripheral lymph node metastasis. Therefore, in our study, we aimed to evaluate the contribution of this technique to diagnosis in patients with pulmonary lesions and to detect recurrence in patients with previously known malignant diseases.

In the classical fine-needle aspiration cytology (FNAC) method, trauma caused by the negative pressure in the syringe and hemorrhagic-degenerative sampling may cause the deterioration of cell quality. In contrast, the non-aspiration needle-sampling technique minimizes tissue damage and collects cells into the needle with the cutting and scraping effect of the advancing tip without applying negative pressure [2]. Many studies in the literature have evaluated the FNAC and FNNAC techniques comparatively and shown that FNNAC is advantageous in terms of reducing the dilution of tumor cells with blood by eliminating the negative suction pressure used in FNAC [3,6,11–16]. Unlike studies showing the superiority of FNNAC over traditional FNAC sampling, it has also been argued that the fine-needle sampling technique used for diagnosis can be left to the personal discretion of the person applying it [17]. Adequate and high-quality tissue supply is crucial for the cytopathologist, and FNNAC allows the cytopathologist to evaluate a precise volume of high cellular samples.

Invasive diagnostic procedures, such as bronchoscopy, mediastinoscopy, and image-guided biopsy are traditionally used in the diagnosis and staging of lung cancer. However, if lymph nodes are detected in neck ultrasound, tomography or positron emission tomography-computed tomography are palpable on physical examination; performing FNAC from these lymph nodes is beneficial for both staging and cytological diagnosis and shows more noninvasive features compared to other procedures. Since the method is fast and easy to perform, it provides the opportunity for an urgent cytological evaluation; therefore, it ensures the optimal use of aspiration material and the rapid identification of patients who require further sampling or additional investigation [18]. In our study, 64% of the cases were first diagnosed with a cytological examination, while FNNAC was performed for the evaluation of progressive disease in the remaining cases. In our study, all diagnostic methods considered to be appropriate for pulmonary lesions were already undertaken, which allowed for the evaluation of the efficacy of FNNAC in confirming these diagnoses and determining whether it contributed to the diagnosis/recurrence/progression stages. Most of our cases were accepted to be in the metastatic stage, and it was once again emphasized that FNNAC was beneficial for both staging and cytological diagnosis. We consider that when the sensitivity and negative predictive values are used together, the FNNAC method can be beneficial at the initial diagnosis stage of the procedure due to its complication-free and low-cost nature, and it is also more appropriate in cases of recurrent, especially in debilitated patients or those that do not accept advanced invasive procedures.

The localization and tissue characteristics of the lesion from FNNAC is applied is one of the factors affecting the diagnostic success of this procedure. In a study comparing the quality of material taken during the procedure, cellularity of smear and amount of blood, it was suggested that FNNAC was less traumatic, more cost effective, and more reliable than FNAC in liver, orbital and thyroid lesions. While better cellular efficiency was obtained from lymph nodes, especially in children, it was argued that this was not recommended for cystic, bony or fibrous lesions [2,15]. There are many studies on performing FNNAC from supraclavicular lymph nodes [19–23]. In our study, the most common localization in which FNNAC was performed was supraclavicular lymph nodes, and it was observed that FNNAC performed from the peripheral lymph node was more successful in diagnosis than the procedure performed from the peripheral pulmonary lesion. This can be explained by the easier accessibility and cellular features of lymph nodes. In a study conducted by Cagle et al. with FNAC, the rate of insufficient sampling was found to be 10% [24]. In our study, this rate was determined as 7.1%. In a study in which lymph nodes were sampled, it was suggested that there was no statistically significant difference between the two techniques in terms of cellularity adequacy. The authors suggested that FNNAC was a better choice considering its additional advantage of less blood staining, especially in lymphadenopathies associated with malignancies and in necrotizing lesions such as tuberculosis [2]. In a study comparing FNAC and FNNAC, especially in terms of their efficacy in staging, it was reported that insufficient sampling might occur in small tumors compared to large tumors, while in advanced large tumors, edema and sclerosis could also affect the diagnostic success of the method. It can be considered that tumoral lesions invading the chest wall may be harder than lymph nodes, and the spontaneous entry of the material into the needle may not be as easy as in a lymph node. In this case, choosing a cutting needle for chest wall lesions seems more appropriate to reach a diagnosis in a single attempt.

In our study, the diagnosis of malignancy was confirmed in most of our patients, and the rate of FNNAC confirming diagnosis was found to be 78.57%. In cases with false negativity, the necrotic or degenerative feature of the lesion suggests that this failure may have been due to reasons such as taking tissue samples containing normal/reactive elements. Some studies reported the need to repeat the FNAC procedure a second time in such patients [18]. In our study, the histopathological confirmation of the main lesion or lymph nodes was already performed by invasive diagnostic methods. The diameter of the lymph node, cellular content, or hemorrhagic-degenerative feature of the sample obtained may have led to a failure in making a diagnosis from the first lymph node, in such cases, complete removal of the same or different lymph node excision can provide more effective tissue sampling than performing a second biopsy on the same lymph node. Studies have also reported that diagnostic difficulties may be experienced in adenocarcinoma cases with focal bronchoalveolar carcinoma features [25]. In our study, five of the seven cases that were evaluated as false-negative had PET CT uptake of the tissues sampled, while the diagnosis of malignancy was confirmed by PFS and EBUS in two patients with lymph nodes nonreported with pathological involvement in PET CT. Therefore, if an infection diagnosis is not primarily considered and if there is a high suspicion of clinical and radiological malignancy, false-negative results should definitely be reevaluated, and more samples should be taken.

The high rate of true positivity of FNNAC can also minimize the complication rate by reducing the number of many invasive diagnostic methods. In addition, this provides convenience and improvements in the evaluation of pulmonary lesions and offers time and cost-efficiency. Patients diagnosed with FNNAC also have a reduced need for highly complicated diagnostic procedures, such as surgical biopsy. Furthermore, in patients with a high risk in terms of invasive procedures or in cases where an invasive procedure is contraindicated, FNNAC from accessible lung lesions, metastatic lesions, or peripheral lymph nodes is not only an easy-to-apply but also safe method in this patient group. It also provides an advantage that it is less costly than invasive methods. Considering the ongoing pandemic conditions, reaching a diagnosis with FNNAC in a short time presents as a great advantage. In our study, no complications were observed except for a case with a mild subcutaneous hematoma, which is supported by the literature data.

In conclusion, in cases with malignant pulmonary lesions, FNNAC performed from the peripheral lymph node presents as a cost effective and safe method for diagnosis and staging, especially in patients that are not suitable for advanced invasive procedures, as well as in the detection of recurrence in those followed up for any malignancy, due to its low complication rate, easy application, and minimally invasive nature.

## Figures and Tables

**Table 1:** Clinical characteristics of the patients.

Characteristics	n
Age, X ± SD (min-max) years	63.9 ± 9.1 (38–80)
Male/female	54/2
Final diagnosis, n	
**Malignant**	
Lung cancer	
*Adenocarcinoma*	16
*Small cell carcinoma*	15
*Squamous cell carcinoma*	10
*Non-small cell carcinoma*	2
*Other*^a^	5
Other malignancies^b^	3
Mesothelioma	3
**Benign**	
Tuberculosis	1
Fibrinous pleuritis	1
Stage^c^, n	
Stage 3	10
Stage 4	44

SD: Standard deviation.

a Other: neuroendocrine carcinoma (n = 2), non-neuroendocrine poorly differentiated carcinoma (n = 1), mixed-type (small+adenocarcinoma) carcinoma (n = 1), clinical-radiological lung cancer (n = 1).

b Other malignancies: renal cell carcinoma (n = 1), lymphoma (n = 1), laryngeal cancer) (n = 1).

c Two cases were excluded from staging due to their benign diagnoses.

**Table 2:** Distribution of lesions from which FNNAC was applied and the number of patients accurately diagnosed with FNNAC.

Type of lesion	Patients that underwent FNNAC (n)	True positivity and negativity of malignancies (n)
Peripheral lymph node		
*Supraclavicular lymph node*	38	32
*Cervical lymph node*	7	6
*Axillary lymph node*	3	2
Peripheral lesion	4	2
Chest wall lesion	4	2
Total	56	44

FNNAC: fine-needle non-aspiration cytology.

**Table 3:** Clinicopathological characteristics of cases with false-negative FNNAC results.

No.	Final diagnosis	FNNAC tissue	PET-CT SUV_max_value of FNNAC tissue	Confirmed diagnosis method
1	Mesothelioma	Peripheral lesion	10.6	TTFNA
2	Adenocarcinoma	Peripheral lesion	6.6	TTFNA
3	Squamous cell carcinoma	Axillary lymph node	5.2	TTFNA
4	Lymphoma	Supraclavicular lymph node	24.5	TTFNA
5	Metastatic malignant pleural fluid	Supraclavicular lymph node	Not reported	PFC
6	Adenocarcinoma	Supraclavicular lymph node	Not reported	EBUS, PFC
7	Adenocarcinoma	Supraclavicular lymph node	9.9	EBUS

FNNAC: fine-needle non-aspiration cytology; PET-CT SUV_max_: positron emission tomography-computed tomography maximum standardized uptake value; TTFNA: transthoracic fine-needle aspiration; PFC: pleural fluid cytology, EBUS: endobronchial ultrasound-guided lymph node biopsy

**Table 4:** Comparative accurate diagnosis rates of FNNAC according to target tissue.

Target tissue	True positivity and negativity of malignancies n (%)	False negativity of malignancies n (%)	Total
Peripheral lymph node	40 (83.3)	8 (16.7)	48
Peripheral pulmonary lesion^a^	4 (50.0)	4 (50.0)	8
Total	44	12	56

FNNAC: fine-needle non-aspiration cytology.

X^2^ = 4.52; p = 0.033

a Peripheral pulmonary lesions represents peripheral pulmonary mass and chest wall mass.
